# Functional Elements of Entrusted Professional Activities for Dental Educators: Protocol for a Scoping Review

**DOI:** 10.2196/74225

**Published:** 2025-06-25

**Authors:** Noraini Abu Bakar, Nurhanis Syazni Roslan, Muhammad Ainul Haq, Yasmin Mustafa Byrnes, Muhamad Saiful Bahri Yusoff

**Affiliations:** 1 Department of Orthodontics Kulliyyah of Dentistry International Islamic University Malaysia Kuantan, Pahang Malaysia; 2 Department of Medical Education School of Medical Sciences Universiti Sains Malaysia Kelantan Malaysia; 3 Centre of Medical Education University of Dundee Dundee United Kingdom; 4 Research and Learning Unit Dar al-Hikmah Library Kuantan International Islamic University Malaysia Pahang Malaysia; 5 Medical Education Research and Innovation Unit Faculty of Medicine and Health Sciences Universiti Putra Malaysia Selangor Malaysia

**Keywords:** dental educators, entrusted professional activities, functional elements, competency-based education, faculty development, assessment, framework

## Abstract

**Background:**

Assessing the competencies of health profession educators presents significant challenges, leading to the suggestion of implementing Entrustable Professional Activities (EPAs) as a potential solution. EPAs refer to the authority granted to individuals to perform tasks with a specified level of trust and competence. The concept of EPAs emerged from the complexities of competency-based medical education. This scoping review highlights the emerging yet underdeveloped field of EPAs for dental educators. Despite their established utility in medical education, the concept remains relatively novel for dental educators, with limited literature and inconsistent application across studies.

**Objective:**

This paper presents a scoping review protocol aimed at systematically elucidating the extent, range, and nature of the literature on EPAs for dental educators, as evaluated in the included resources. This review seeks to answer the following research question: what are the key functional elements of EPAs for dental educators as described in the existing literature?

**Methods:**

This scoping review will be conducted in accordance with the framework outlined by Arksey and O’Malley, enhanced by the Joanna Briggs Institute (JBI) guidelines. The literature search will use a 3-step strategy across 5 electronic databases (PubMed, Google Scholar, Scopus, Cochrane Library, and ProQuest), along with additional screening of gray literature. Primary data from relevant studies published from the introduction of EPAs in 2005 through 2024 will be analyzed. This review will include peer-reviewed literature focused on EPAs, specifically those involving dental educators in undergraduate, postgraduate, or continuing education contexts. Studies of any design (qualitative, quantitative, or mixed methods), as well as conceptual papers and reviews, will be considered. Only English-language studies will be included. The literature search process will involve a librarian, while study selection and evidence charting will be conducted by 4 independent reviewers. These reviewers will independently screen titles, abstracts, and full texts against the inclusion criteria. Data will be charted using a standardized extraction form and analyzed thematically.

**Results:**

Findings will be presented in both tabular and narrative formats, highlighting the range, characteristics, and key components of EPAs relevant to dental educators. In addition, these findings will be mapped to the competencies of dental educators. This review is estimated to be completed within 6 months and aims to identify gaps for future research, ultimately supporting the advancement of competency-based dental education. The results of this review will serve as a foundation for a larger study focused on developing a framework of EPAs specifically tailored for dental educators.

**Conclusions:**

The identification of EPAs and their themes provides an initial framework that can guide the development of more structured, competency-based academic roles for dental educators. A tailored EPA framework holds promise not only for clarifying faculty expectations and enhancing performance assessment but also for supporting faculty development.

**International Registered Report Identifier (IRRID):**

DERR1-10.2196/74225

## Introduction

### Background

Educators in the health professions often bear significant responsibilities as they must fulfill dual roles as both health care providers and educators. Health professions educators are usually trained in their own clinical specialty and receive very little training to be equipped as educators [1]. The educators are the primary instrument for knowledge transfer; however, little emphasis has been placed on the capabilities or competencies of the health professions educators. The question that remains is as follows: how can we assess educators’ competency?

### EPAs for Health Professions Educators

The competency of educators in Health Professions Education (HPE) has often been undervalued. However, in recent years, an increasing number of medical education professionals have begun to underscore the importance of ensuring educator competency. Concerns have been raised regarding the evidence that supports the assertion that educators are adequately prepared to guarantee the safety and high quality of health care systems, which is essential for maintaining patient trust [2-4]. Despite the foundational work that has been undertaken in this area, assessing the competencies of health professions educators presents significant challenges. This has led to the suggestion of the implementation of Entrustable Professional Activities (EPAs) as a potential solution.

The concept of EPAs emerged from the complexities of competency-based medical education (CBME) [2]. EPAs were introduced with the concept of trust as a central element for effective execution. Initially proposed by Olle Ten Cate in 2005, EPAs are defined as tasks or responsibilities that can be entrusted to a trainee once they have achieved sufficient, specific competence to permit unsupervised practice [5]. The terms “Entrustable” and “Entrusted” are related and have often been used interchangeably in the literature. Therefore, to avoid confusion, the term “Entrustable” will be used exclusively from this point onward.

EPAs represent units of professional practice that are independently executable, observable, and measurable in both their process and outcomes, making them suitable for entrustment decisions [6]. In short, EPAs serve as a means to translate competencies into practical application. EPAs are hoped to link the actual teaching activities and support the development of teaching [1].

### EPAs in Dentistry and Dental Educators

Dentistry, as a hands-on clinical program, would significantly benefit from a competency-based approach; thus, EPAs are regarded as a valuable addition to dental training curricula. The concept of EPAs in dentistry has been addressed in numerous publications; however, significant emphasis has largely been placed on students rather than on the capabilities and competencies of educators. It is now imperative to shift the focus of EPAs toward educators, as they play a crucial role as a vital link between the curriculum and the students. This shift is essential to ensure a high-quality educational experience for all learners while maintaining accountability and consistency [7].

There is a growing recognition that for the dental health profession to fulfill its responsibility of training competent practitioners, emphasis must also be placed on the competencies of dental educators. In 1999, the American Association of Dental Schools reported that the quality of dental students’ educational experiences is critically dependent on having an adequate number of dedicated faculty with expertise in both content and teaching methodologies [8]. Furthermore, in 2010, the UK Committee of Postgraduate Dental Deans and Directors (COPDEND) established guidelines for dental educators that can be used in their employment, development, and management [9].

There is, however, a notable deficiency in published evidence regarding EPAs for dental educators. Most existing literature on EPAs primarily focuses on student assessments, particularly within postgraduate dental training [10]. A scoping review conducted in 2023 examined EPAs within the broader framework of dental education. The review primarily focused on the development of EPAs for undergraduate and postgraduate dental students, as well as the evaluation of EPAs in general dentistry, without addressing the perspectives of dental educators [11]. This proposed scoping review aims to specifically focus on the literature related to EPAs for dental educators. The findings of this review will establish a foundation for a larger study designed to develop a framework of EPAs tailored specifically for dental educators. While there is a notable gap in the literature on this topic, some evidence does exist. Therefore, a scoping review is essential to systematically search for and report the current findings on EPAs for dental educators. This effort is intended to provide resources for dental educators to incorporate EPAs into training contexts and facilitate faculty development in assessing and enhancing the skills of their educators. This approach also aligns with the skill-based methodologies favored by most universities.

### Objectives and Research Questions

This scoping review aims to explore the breadth and extent of evidence regarding EPAs for dental educators. The review will address this 1 research question: what are the key functional elements of EPAs for dental educators as described in the literature?

## Methods

### Study Design

This scoping review protocol will follow the methodological framework outlined by Arksey and O’Malley [12] and in accordance with the Joanna Briggs Institute (JBI) guidelines for scoping reviews [13]. Any modifications to the methodological approach will be documented and described in the final scoping review report, according to the PRISMA-ScR (Preferred Reporting Items for Systematic Reviews and Meta-Analyses extension for Scoping Reviews) guidelines [14]. The adoption of 2 frameworks enhances the credibility and trustworthiness of the review through systematic procedures, while also allowing for adaptability based on emerging insights, thereby meeting high academic standards.

### Review Team

The review team consists of 2 qualified medical education experts, each holding a doctorate in medical education and possessing over 5 years of experience in medical education teaching and conducting scoping reviews (MSBY and NSR). Furthermore, the team includes 2 experienced dental educators: one is the lead of the dental education pathway in a renowned medical education center (MAH), while the other currently pursuing a PhD in medical education (NAB). A qualified librarian is also engaged to support the development and refinement of the search strategy to ensure comprehensiveness and systematic retrieval of relevant literature across selected databases (YMB).

### Inclusion Criteria

#### Types of Articles

Given the terms “entrusted” and “entrustable” are related, ensuring no articles are overlooked, this scoping review will examine published primary and secondary research that describes both the Entrusted and Entrustable Professional Activities for dental educators globally, using 5 databases (PubMed, Google Scholar, Scopus, Cochrane Library, and ProQuest). Resources outside of these databases will be excluded from the review.

#### Concept

This scoping review will encompass resources to capture the evidence, scope, and range of EPAs for dental educators. This review will specifically focus on the EPAs pertinent to dental educators. Dental educators in this context will include individuals involved in teaching at all levels of dental education, including both undergraduate and postgraduate programs, regardless of geographical background. In addition, this review will include articles that explore the various elements related to EPAs, including domains, characteristics, factors, skills, or themes that shape the EPAs essential for effective dental educators. Resources that do not explicitly report on EPAs will be excluded from this review.

#### Context

The context of this review will encompass the elements, characteristics, domains, or any other related themes that build or describe the EPAs for dental educators irrespective of their teaching level. This includes educators for both undergraduate and postgraduate education including clinical specialty training.

The components of the EPAs for dental educators are grounded in the competencies being assessed, which include educational theories and principles, modes of education, learner issues, educational materials, instructional design, curriculum matters, evaluation, research, management, quality assurance, patient care and health care systems, and professionalism [15]. Resources that do not pertain to the themes of the EPAs and articles unrelated to dental educators will be excluded. The inclusion and exclusion criteria are summarized in Textbox 1 and definitions are provided in Textbox 2.

**Inclusion and exclusion criteria used during the screening of the population (dental educators), concept (EPAs), and context (element, domain, item, skills, and characteristics) examined in the scoping review.**

**Inclusion criteria:**

- Population
  Dental educators or dental lecturers.
  Including either undergraduate- or postgraduate-level educators.
  Including both private and public education institutions.
- Concept
  Entrustable professional activities.
- Context
  Element, standard, domain, item, skills, or characteristics of dental educators.
- Other
  Published in the English language.
  Study design: all study types.
  Study location: from all geographical locations.
  Published between 2005 and 2024.
  Full text.

**Exclusion criteria:**

- Population
  Nonhealth, nonmedical, and nondental related educators.
- Other
  Published in a language other than English.
  Non-full text.

**Definition for the Population, Concept, Context, and characteristics for this scoping review.**

- Entrustable professional activities: tasks or responsibilities (to be mastered or certified) that can be entrusted to a trainee, once sufficient, specific competence is reached to allow unsupervised execution [6].
- Dental educator: a professional who teaches dental students and other dental professionals through formal clinical teaching in dental schools including through outreach programs [7].
- Element: one of the parts of something that makes it work, or a quality that makes someone or something effective (Cambridge Dictionary).

#### Sources

This scoping review will encompass both quantitative and qualitative research and is not limited to primary studies. Since EPAs are relatively new to dentistry, gray literature is considered a crucial source of information [15]. Therefore, to ensure that no significant data are overlooked, both primary research and secondary reviews—including, but not limited to, narrative reviews, scoping reviews, systematic reviews, and meta-analyses—as well as published gray literature limited to conference proceedings, theses and dissertations, working papers, preprints, and protocols relevant to the EPAs of dental educators, will be systematically searched.

As EPAs were first introduced in 2005, the review will only include articles published in English between January 1, 2005, and October 1, 2024. Unpublished literature, websites, and blog posts will not be considered in this review to maintain the authenticity and reliability of the data.

### Search Strategy

This review will be conducted using a 3-phase search strategy based on the recommendations of the JBI Scoping Review Guidelines [13]. The initial keywords will be identified and selected from the titles and index terms of relevant reviews. These keywords will be derived from the MeSH (Medical Subject Headings) and Education Resources Information Center (ERIC) databases and will be tested with various search terms using Boolean combinations. The search terms will be refined and adapted accordingly after multiple test searches. A qualified librarian (YMB) was consulted to assist in the development of the search strategy, including the identification of relevant databases, refinement of search terms and Boolean operators, and the adaptation of search strings for each database. The librarian also contributed to the peer review of the final search strategy to ensure sensitivity and comprehensiveness. The initial search will be based on the Table 1 below.

**Table 1:** Table of the key concepts, free text terms, and controlled vocabulary terms.

	Concept 1	Concept 2
Key concepts	Entrustable professional activities	Dental educators, dental education, or dentistry
Free-text terms, natural language terms, or author keywords (from papers)	Entrustable professional activities Entrusted professional activity Entrustable professional activity	Dental educator Dental educators Dental education Dentistry
Controlled vocabulary terms or subject terms	Not applicable	Index terms, MeSH^a^, or Emtree MeSH (education, dental, and dentistry)

^a^MeSH: Medical Subject Headings.

The initial keyword search terms will be based on a few key papers and further refined accordingly after a few searches. Keywords identified as (“Entrusted Professional Activities” OR “Entrustable Professional Activities” OR “Entrustable profession* activit*”) AND (“Dental Educator*” OR “Dental Education” OR Dentistry), with a time limit of 2005 to 2024.

A second search using all identified keywords and index terms will be performed across 5 databases, namely Scopus, Google Scholar, PubMed, Cochrane Library, and ProQuest. Reference lists of eligible studies will be back searched as the third phase. The findings will be summarized in tabular form and a narrative synthesis.

### Selection Process

The record selection will adhere to the predefined inclusion criteria with the search term monitored by the librarian (YMB). All identified sources will be exported into Microsoft Excel and duplicates will be removed. Once the reviewers are familiar with the selection process, the titles, abstracts, and full-text articles of the included records will be screened according to the inclusion criteria. The selection process will be conducted independently by 2 researchers (NAB and NSR), with any disagreements addressed through the involvement of the other 2 reviewers (MSBY and MAH) and further discussion, if necessary. Records that do not meet the inclusion criteria will be excluded from this study, and the reasons for exclusion will be documented. The search profile of the selection process will be reported using the PRISMA-ScR flow diagram Multimedia Appendix 1 [14].

### Data Extraction, Analysis, and Presentation of the Results

A standardized data extraction form is developed based on the JBI guideline as in Multimedia Appendix 2 [13]. The following data will be extracted from each article:

1. Title
2. Authors and year of publication
3. Country or geographical area
4. Study design, tool, or intervention
5. Participant categories (dental educators for undergraduate or postgraduate or both)
6. Elements or characteristics of EPAs
7. Key findings
8. Other relevant information and remarks

This data extraction will be performed independently by 2 reviewers to mitigate the risk of error. To ensure the reliability and consistency of the data extraction process, the reviewers will discuss their extraction strategies and pilot the process by extracting data from 5 records. Transparent reporting will be implemented and duplication of information from primary literature and reviews will be avoided by including a clear list of primary studies to flag duplicates. In addition, the reviewers will collaborate to discuss the outcomes of the data extraction. Any modifications in the data extraction form following the piloting will be reported.

The extracted data will be presented in tabular form and described using descriptive statistics. A thematic approach will be used using NVivo software (Lumivero) for qualitative analysis. The data will be thematically organized according to the elements of the EPAs for dental educators and other related factors. The process will begin with open coding of relevant text (eg, descriptions of EPAs, functional elements, and implementation strategies), followed by a grouping of codes into broader categories and ultimately, into meaningful themes. A narrative summary of each element will be provided, and the elements of the EPAs for dental educators will be mapped to their associated issues or any relevant emerging theme identified during the review. Themes will be refined through team discussions to ensure credibility and consistency. The final thematic map will be used to answer the main research question by identifying key concepts and applications of EPAs for dental educators.

### Quality Assessment

A quality assessment of the included articles will be conducted using the tool developed by Kmet and colleagues [16]. While this step is not mandatory in a scoping review, its inclusion will enhance the analysis. This tool provides standardized criteria for evaluating the methodological quality of primary research papers across various fields. The quality assessment will help identify potential sources of bias, assess the rigor of study designs, and provide insights into the overall quality of the evidence. A total of 2 reviewers will independently carry out the quality assessment, with any discrepancies resolved through discussion or consultation with the third and fourth reviewers.

**Ethical Considerations**

This review is classified as a secondary research study, concentrating on the existing literature, therefore, ethical approval is not required.

## Results

### Overview

Data collection started in October 2024 and is expected to conclude in May 2025 and expected results to be published by the end of 2025.

### Expected Outcome

This scoping review aims to offer these outcomes:

1. Provide a comprehensive and structured overview of the literature regarding EPAs for dental educators.
2. Identify the key functional elements of EPAs for dental educators from the available evidence.
3. Contributes to the existing literature on EPAs for dental educators.

## Discussion

### Principal Findings

This scoping review sought to systematically map the existing literature on EPAs relevant to dental educators. Although a limited number of articles are expected, we anticipate identifying a potential number of EPAs that can be mapped to overarching themes. The limited number of studies reflects a significant gap in scholarly focus on defining and applying EPAs for dental educators.

### Comparison to Prior Work

While EPAs have gained considerable traction in medical education, particularly in postgraduate training for physicians and other health professionals, their application in dentistry especially dental educators remains underexplored. Prior studies in medical education have emphasized EPAs as a bridge between broad competencies and day-to-day clinical tasks, facilitating both curriculum design and assessment of workplace readiness. Similar principles could be beneficial for dental educators, particularly in aligning educational tasks with observable and measurable outcomes.

The reviewed articles primarily adapted frameworks from medical education, often with minimal contextualization for the unique scope of dental academic responsibilities. This highlights a critical gap: dental educators’ roles-spanning teaching, clinical supervision, research, and administrative duties, demand a tailored EPA framework that reflects the distinct characteristics of dental academia. This review fills this gap by consolidating available evidence and categorizing EPAs into meaningful themes, providing a starting point for further validation and adaptation in dental contexts.

### Strengths and Limitations

A key strength of this review lies in its rigorous methodology, guided by the Arksey and O’Malley [12] framework and enhanced through the JBI guidelines. The comprehensive search strategy across multiple databases and inclusion of gray literature ensures a broad capture of relevant materials. The thematic synthesis of the findings offers a structured way to interpret the scattered evidence base. The review also incorporated multiple expert reviewers, including a librarian, to ensure a high-caliber search.

However, several limitations should be acknowledged. First, the small number of included studies and the relatively recent emergence of EPAs in dental education limit the generalizability of the findings. Second, many articles lacked in-depth descriptions of how EPAs were developed, implemented, or assessed, making it difficult to compare methodologies or outcomes across studies. Third, the variation in terminology and conceptual understanding of EPAs may have resulted in relevant studies being excluded despite using different nomenclature.

These limitations suggest that findings should be interpreted with caution. Future research should aim to clarify the operational definition of EPAs for dental educators and develop standardized processes for their design, implementation, and assessment.

### Practical Implications

Despite the limited evidence, the review provides several important implications for dental education. The identified EPAs can inform curriculum development for academic training programs, especially for junior faculty members or those undergoing academic leadership development. EPAs also have potential utility in academic performance evaluation, faculty development, and the mentoring of early-career educators by clearly defining what tasks can be entrusted at various levels of competence.

Moreover, the adoption of EPAs in dental education could enhance clarity in role expectations, promote accountability, and facilitate structured feedback—all critical elements for professional development in academic settings.

### Future Directions

To build upon this foundation, further research is needed to:

1. Develop and validate a standardized set of EPAs specifically tailored for dental educators.
2. Engage stakeholders including faculty, administrators, and students in cocreating and refining EPA frameworks to ensure contextual relevance.
3. Explore the feasibility of implementing EPAs in academic promotion, continuing professional development, and interprofessional education.
4. Investigate how EPAs can support transitions in faculty roles, such as from clinician to educator or from junior to senior academic positions.

Longitudinal studies and implementation research could further clarify the impact of EPAs on educational quality, faculty satisfaction, and institutional outcomes.

### Dissemination Plan

The findings of this review will be disseminated through publication in peer-reviewed journals and presentations at conferences related to health professions education and dental education. In addition, summaries will be shared with academic leadership and faculty development units to inform ongoing and future initiatives related to academic competency frameworks.

### Conclusion

The identification of EPAs and their themes provides an initial framework that can guide the development of more structured, competency-based academic roles for dental educators.

The findings underscore the need for further empirical research to refine and validate EPAs specific to the dental education context. A tailored EPA framework holds promise not only for clarifying faculty expectations and enhancing performance assessment but also for supporting faculty development and succession planning. By advancing the understanding and application of EPAs in dental education, this study lays a foundational step toward more systematic, accountable, and outcome-based academic practice.

## Appendix

Multimedia Appendix 1 PRISMA-ScR checklist.

Multimedia Appendix 2 Data extraction form.

## Abbreviations

CBME: competency-based medical education
COPDEND: Committee of Postgraduate Dental Deans and Directors
EPA: Entrustable Professional Activity
ERIC: Education Resources Information Center
HPE: Health Professions Education
JBI: Joanna Briggs Institute
MeSH: Medical Subject Headings
PRISMA-ScR: Preferred Reporting Items for Systematic Reviews and Meta-Analyses extension for Scoping Reviews

## Data Availability

The datasets and/or any supplementary material used in this scoping review will be available from the corresponding author upon request.

## References

[1] van Bruggen L, van Dijk EE, van der Schaaf M, Kluijtmans M, Ten Cate O (2022). Developing entrustable professional activities for university teachers in the health professions. Med Teach.

[2] Dewey CM, Jonker G, Ten Cate O, Turner TL (2017). Entrustable professional activities (EPAs) for teachers in medical education: has the time come?. Med Teach.

[3] Wolcott MD, Quinonez RB, Ramaswamy V, Murdoch-Kinch CA (2020). Can we talk about trust? Exploring the relevance of "Entrustable Professional Activities" in dental education. J Dent Educ.

[4] Chartash D, Rosenman M, Wang K, Chen E (2022). Informatics in undergraduate medical education: analysis of competency frameworks and practices across North America. JMIR Med Educ.

[5] Ten Cate O (2013). Competency-based education, entrustable professional activities, and the power of language. J Grad Med Educ.

[6] Ten Cate O (2013). Nuts and bolts of entrustable professional activities. J Grad Med Educ.

[7] Orr G, Porter S, Sharp I (2013). Standards for Dental Educators COPDEND.

[8] Lyon LJ (2009). Developing teaching expertise in dental education. ERIC Home.

[9] Bullock AD, Firmstone VR, Falcon HC (2010). Developing guidelines for postgraduate dental educators in the UK. Br Dent J.

[10] Kelly GM, Roberts A, Lynch CD (2022). A literature review: entrustable professional activities, an assessment tool for postgraduate dental training?. J Dent.

[11] Ehlinger C, Fernandez N, Strub M (2023). Entrustable professional activities in dental education: a scoping review. Br Dent J.

[12] Arksey H, O'Malley L (2005). Scoping studies: towards a methodological framework. Int J Soc Res Methodol.

[13] Peters MDJ, Godfrey C, McInerney P, Khalil H, Larsen P, Marnie C, Pollock D, Tricco AC, Munn Z (2022). Best practice guidance and reporting items for the development of scoping review protocols. JBI Evid Synth.

[14] Moher D, Liberati A, Tetzlaff J, Altman DG, PRISMA Group (2009). Preferred reporting items for systematic reviews and meta-analyses: the PRISMA statement. PLoS Med.

[15] Chuenjitwongsa S, Bullock A, Oliver RG (2018). Roles and competences for educators of undergraduate dental students: a discussion paper. Eur J Dent Educ.

[16] Kmet LM, Lee RC, Cook LS, Alberta Heritage Foundation for Medical Research (2004). Standard quality assessment criteria for evaluating primary research papers from a variety of fields.

