# Supplementary material for: Functional Elements of Entrusted Professional Activities for Dental Educators: Protocol for a Scoping Review
Source: JMIR Res Protoc. 2025 Jun 25;14:e74225. doi: 10.2196/74225 (PMC12242055; doi:10.2196/74225)
Supplement: Multimedia Appendix 2 [file resprot_v14i1e74225_app2.docx]

Appendix 1

Data Extraction Form

| Title of articles | Authors | Year of publication | Study Type | Geographical distribution (state the study site - institution, state, country) | Participants Age (range) | Participants Category (Level:  Undergraduates, Postgraduates)  Clinical/BMS | Nature/scope/  range of EPA | Element/  Character/Factor of EPA | Remarks |
| --- | --- | --- | --- | --- | --- | --- | --- | --- | --- |
|  |  |  |  |  |  |  |  |  |  |
|  |  |  |  |  |  |  |  |  |  |
|  |  |  |  |  |  |  |  |  |  |
